# The catalytic and kinetic characterization of *Bacillus subtilis* MK775302 milk clotting enzyme: comparison with calf rennet as a coagulant in white soft cheese manufacture

**DOI:** 10.1186/s43141-023-00513-w

**Published:** 2023-05-17

**Authors:** Hala R. Wehaidy, Mohamed A. Abdel-Naby, Adel M. M. Kholif, Mostafa Elaaser, Wafaa K. Bahgaat, Walaa A. Abdel Wahab

**Affiliations:** 1 Chemistry of Natural and Microbial Products Department, National Research Centre, Dokki, Giza, Egypt; 2 Dairy Sciences Department, National Research Centre, Dokki, Giza, Egypt

**Keywords:** *Bacillus subtilis* MK775302, Milk clotting enzyme kinetics, UF-white soft cheese

## Abstract

**Background:**

Calf rennet is considered the traditional source of milk clotting enzyme (MCE). However, increasing cheese consumption with decreasing the calf rennet supply had encouraged the quest for new rennet alternatives. The purpose of this study is to acquire more information about the catalytic and kinetic properties of partially purified *Bacillus subtilis* MK775302 MCE and to assess the role of enzyme in cheese manufacture.

**Results:**

*B. subtilis* MK775302 MCE was partially purified by 50% acetone precipitation with 5.6-fold purification. The optimum temperature and pH of the partially purified MCE were 70 °C and 5.0, respectively. The activation energy was calculated as 47.7 kJ/mol. The calculated *Km* and *Vmax* values were 36 mg/ml and 833 U/ml, respectively. The enzyme retained full activity at NaCl concentration of 2%. Compared to the commercial calf rennet, the ultra-filtrated white soft cheese produced from the partially purified *B. subtilis* MK775302 MCE exhibited higher total acidity, higher volatile fatty acids, and improved sensorial properties.

**Conclusions:**

The partially purified MCE obtained in this study is a promising milk coagulant that can replace calf rennet at a commercial scale to produce better-quality cheese with improved texture and flavor.

**Supplementary Information:**

The online version contains supplementary material available at 10.1186/s43141-023-00513-w.

## Background

Rennet (milk clotting enzyme) is an enzyme complex that contains many enzymes: chymosin or rennin (EC 3.4.23.4) and protease which are the major enzymes used in the cheese industry. Moreover, it contains other important enzymes such as pepsin and lipase [1, 2]. Milk clotting enzymes facilitate milk clotting and also control cheese yield, texture, and flavor [3]. Cheese is a milk derivative that is rich in protein, fat, sodium, and calcium. It is produced by adding the starter culture and milk clotting enzyme [4]. The type of milk-clotting enzyme plays a vital role in milk coagulation and cheese maturation as it has a significant impact on the quality of the produced cheese [5].

For a long time, calf rennet extracted from the fourth stomach of young calves (contains rennin or chymosin EC 3.4.23.4) has been used as the most common coagulant in cheese manufacture all over the world. The increased production and consumption of cheese around the world, as well as the scarcity of calf rennet, has prompted researchers to look for other sources of coagulant proteases [6]. These proteases should have high milk clotting activity and low proteolytic activity at the optimum conditions of cheese manufacture. The use of animal-derived coagulants has many drawbacks as they are expensive, and their consumption has been banned for religious or nutritional reasons [7]. Most plant coagulants are unsuitable for cheese making due to the low yield of cheese, the bitter taste, and defects in the flavor of the produced cheese [8]. Milk clotting enzymes from microbial sources have gained great attention due to their various properties, ease of preparation, and reduced cost of production [9]. Bacterial milk clotting enzymes are more preferable compared to fungal enzymes due to lower production costs, greater biochemical diversity, faster cultivation, and easier genetic modification [10]. Many nonpathogenic *Bacillus* species have been used as potential MCE producers [11].

The kinetic stability of an enzyme is an evaluation of its resistance to irreversible inactivation at certain temperature, and it is commonly expressed as the enzyme half-life time (*t*_1/2_) [12]. Good knowledge of enzyme stability is important for its application in biotechnological processes since it provides information about the enzyme’s structure and allows a more cost-effective application strategy. Furthermore, a better understanding of enzyme stability under different working circumstances could aid in improving the efficiency of enzymatic operations [13]. However, very few studies exist concerning the kinetic parameters of milk clotting enzymes.

The present study aims to provide some information about the catalytic and kinetic parameters of the partially purified *B. subtilis* MK775302 MCE and also to evaluate the enzyme in cheese manufacture. The study was conducted by using the partially purified microbial rennet in a comparison with the commercial calf rennet for the manufacture of UF white soft cheese.

## Methods

### Partial purification of *B. subtilis* MK775302 MCE

The crude MCE extract obtained from *B. subtilis* MK775302 [14] was partially purified by fractional precipitation with acetone at different concentrations (25, 50, and 75%). The obtained fractions were dried and assayed for milk clotting activity (MCA). The most active fraction was used for characterization and UF white soft cheese manufacture.

### Assay of milk clotting activity (MCA)

The activity of milk clotting was measured as described by Arima et al. [15]. The skimmed milk was prepared at 12% concentration (dissolved in 0.1-M acetate buffer, pH 5.0) and used as a substrate.

$$\mathrm{MCA }(\text{Soxhlet units}) = 2400/\mathrm{T }\times \mathrm{ S}/\mathrm{E}$$

where S is the volume of skimmed milk (ml), E is the volume of MCE (ml), and T is the clotting time (s).

### Catalytic and kinetic characterization of the purified *B. subtilis* MK775302 MCE

The catalytic and kinetic properties of the partially purified enzyme were characterized. These include determining the optimum operating temperature and pH. These also include studying the enzyme thermal stability and determination of *t*_1/2_, *K*_d_, (*D*), *V*_max_, and *K*_m_ values.

### Optimum temperature

The optimum temperature of MCE was investigated by assaying the partially purified enzyme milk clotting activity at different temperatures (from 40 to 80 °C). The activation energy (*Ea*) was calculated from the slope of the Arrhenius plot according to the following equation:

$$\mathrm{Slope}=-Ea/2.303R$$

where R is the gas constant (8.314 kJ.mol.^−1^)

### Optimum pH

The MCA of the partially purified *B. subtilis* MK775302 MCE was assayed at different pH values (from 5.0 to 7.0 using 0.1-M acetate and 0.1-M phosphate buffer) to determine the optimal pH.

### Thermal stability of *B. subtilis* MK775302-purified MCE

In this experiment, the partially purified enzyme was heated in the absence of substrate in a water bath at different temperatures ranging from 50 to 70 °C. A sample was removed every 15 min and cooled. The residual activity was determined at the optimum reaction conditions, and the activity at zero time was considered as 100%. The deactivation rate constant (*k*_d_) was calculated from the slope when the log of residual activity was plotted against the time of deactivation. The half-life time (*t*_1/2_) and the decimal reduction time (*D*) (defined as the time required for 90% reduction in the initial enzyme activity at a specific temperature) were calculated also.

### Effect of substrate concentration

MCA was determined at different substrate concentrations (60–140 mg/ml) at the optimum assay conditions. The maximum reaction velocity (*V*max) and Michaelis–Menten constant (*K*m) values were determined from the Linweaver-Burk plot.

### Effect of sodium chloride on MCA

The effect of sodium chloride on MCA was examined by incubating the partially purified MCE with different concentrations of NaCl (from 0 to 4%) for 60 min at room temperature. The MCA was then determined [16].

### White soft cheese manufacture

Two batches of UF white soft cheese (using calf rennet and the partially purified *B. subtilis* MK775302 MCE) were manufactured according to El-Shibiny et al. [17] with some changes. The fresh buffalo’s milk was ultra-filtrated and salted with 3% sodium chloride, pasteurized at 72 °C for 15 s and then cooled to 37 °C, and it was divided into two equal portions. *Lactococcus lactis* spp. *lactis* and *Lactococcus lactis* spp. *cremoris* (1% active culture) were used to inoculate both batches. Calcium chloride was added at a concentration of 0.02%. Commercial calf rennet was used to coagulate the first portion (the control sample), and the second portion was coagulated by the partially purified *B. subtilis* MK775302 MCE. The resultant UF white soft cheese samples were instantly packed in plastic cups filled with 3% pasteurized salted permeate and stored at 5 ± 2 °C for 28 days.

### The physical and chemical analysis of UF-white soft cheese

The physical and chemical analysis were carried out for both cheese samples (calf rennet and partially purified MCE cheese) at different storage periods (0, 7, 14, 21, and 28 days). The moisture contents, total nitrogen (TN), and soluble nitrogen (SN) were determined according to AOAC [18]. Total nitrogen and soluble nitrogen were determined by the semi-micro Kjeldahl distillation method. The total acidity (TA) of the soft cheese samples was determined as lactic acid content by titration with 0.1-N sodium hydroxide solution as described by Ling [19]. The total volatile fatty acid (TVFA) was determined according to Kosikowski [20].

### Textural profile analysis (TPA)

TPA was performed according to the method of Glibowski et al. [21] by using three samples for each cheese treatment. The double compression test TA-XT2i texture analyzer (Stable Microsystems, Godalming, UK) was used. Compression tests were used which produced a force versus time plot. The samples were compressed by 30% of their depth.

### Sensory evaluation of UF-white soft cheese samples

The total accessibility score was assigned as 100 points. The produced cheese samples were organoleptically evaluated according to Davis [22]. Fifty points were assigned for flavor, forty points for body texture, and ten points for appearance. All cheese samples were tested by the Dairy Science Department members at the National Research Center at different cold storage periods (0, 7, 14, 21, and 28 days).

### Statistical analysis

The statistical analysis was performed according to the statistical analysis system (SAS) using the general linear model (GLM) of SAS Institute [23]. The results were expressed as means, and the difference between means was tested using Duncan’s multiple ranges at *p* ≤ 0.05.

## Results

### Partial purification of crude *B. subtilis* MK775302 MCE

The fractional precipitation of the crude *B. subtilis* MK775302 MCE with acetone yielded 3 fractions (25, 50, and 75%). The most active enzyme fraction was obtained at 50% concentration with a specific activity of 175.8 U/mg protein and 5.6-fold purification (Table 1). Therefore, this fraction will be used in further studies for characterization and cheese manufacture.

**Table 1:** Partial purification of crude *B. subtilis* MK775302 MCE

Acetone concentration (%)	Protein content mg/fraction	Activity of fraction (U)	Specific activityU/mg protein	Recovered protein (%)	Recovered activity (%)	Fold purification
Culture filtrate	1920	60,000	31.3	100	100	1.00
25	70.38	5532.6	78.6	3.66	9.22	2.5
50	158	27,790	175.8	8.2	46.3	5.6
75	140.52	10,512	74.8	7.32	17.5	2.4

### Catalytic and kinetic characterization of *B. subtilis* MK775302-purified MCE

#### Optimum temperature

**Fig. 1:**
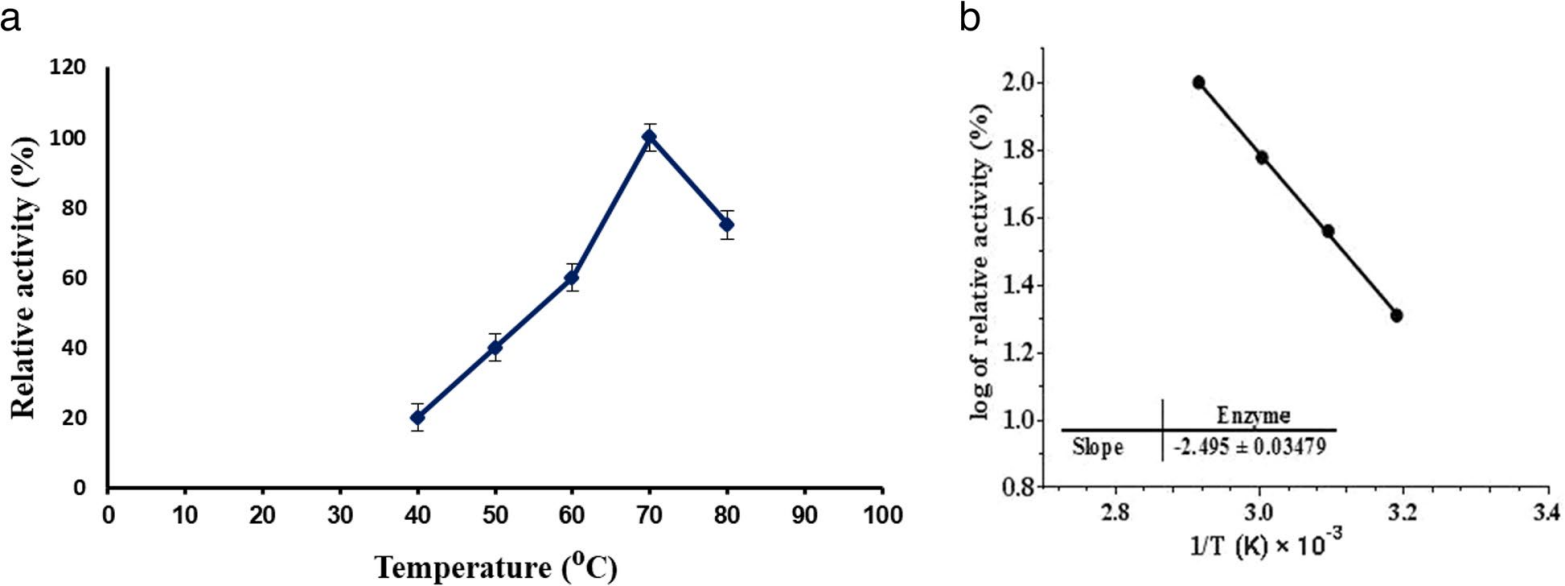
**a**,** b** Effect of temperature on *B. subtilis* MK775302-purified MCE

As represented by Fig. 1 (a, b), the partially purified enzyme exhibited maximum MCA at a temperature of 70 °C. Above this temperature, the MCA was markedly decreased.

#### Optimum pH

**Fig. 2:**
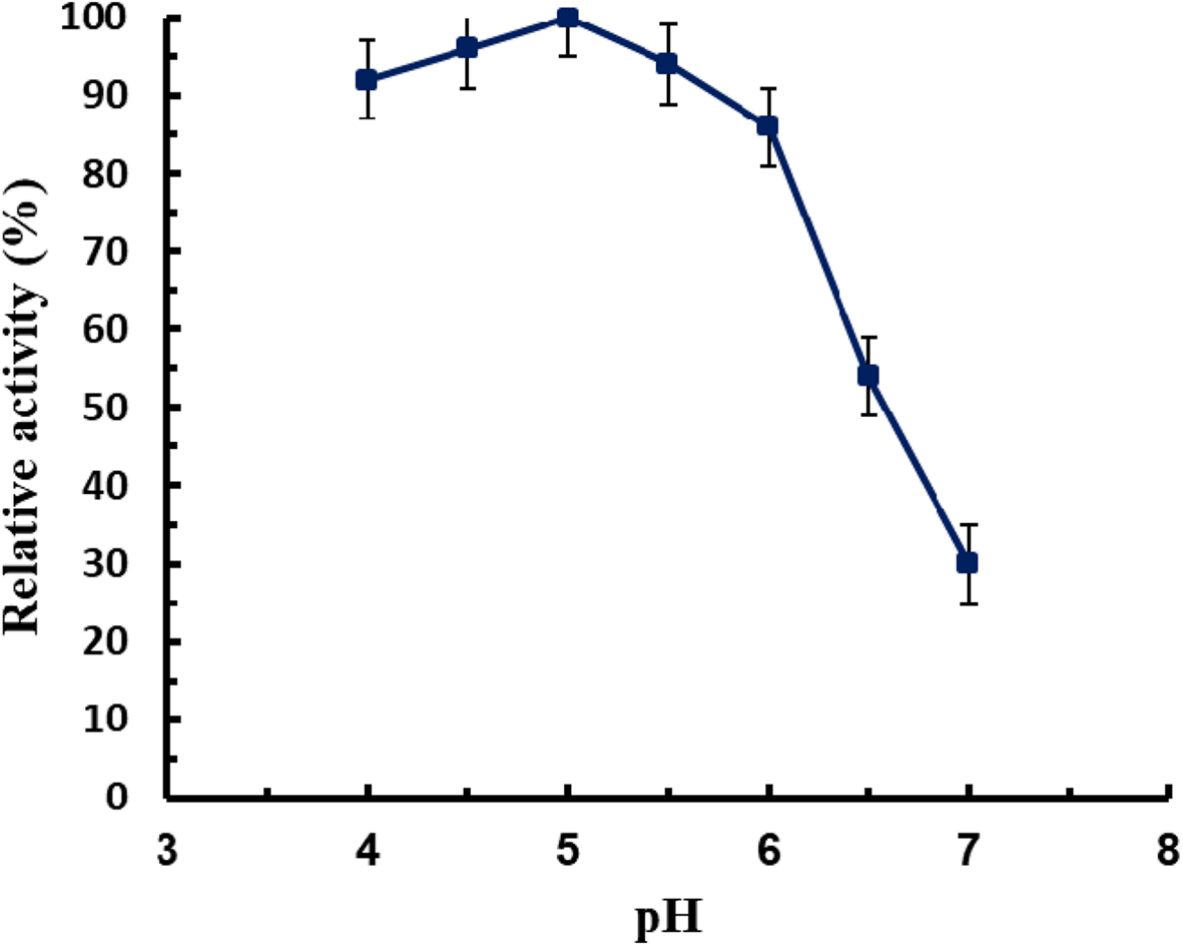
Effect of pH on *B. subtilis* MK775302-purified MCE

The maximum MCA (800 U/ml) was observed at pH 5.0 (Fig. 2). The activity decreased rapidly at milk pH > 6. A higher pH value caused a considerable decrease in enzyme activity.

#### Thermal stability of *B. subtilis* MK775302 purified MCE

**Fig. 3:**
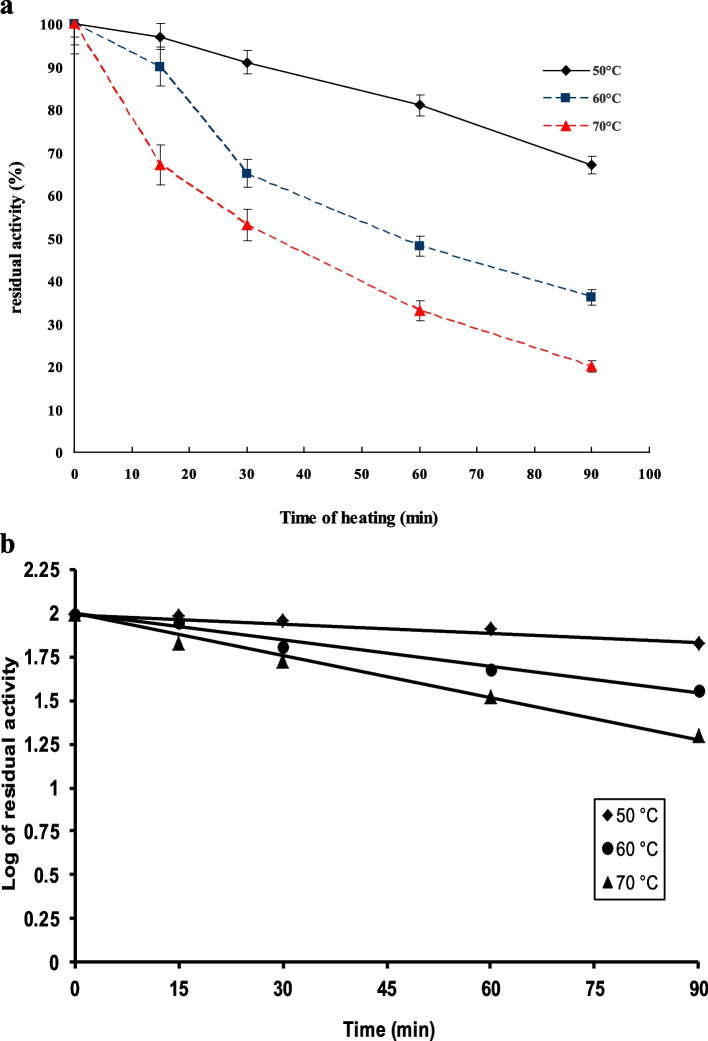
**a** Thermal stability of *B. subtilis* MK775302-purified MCE. **b** Log of residual activity as a function of time

**Fig. 4:**
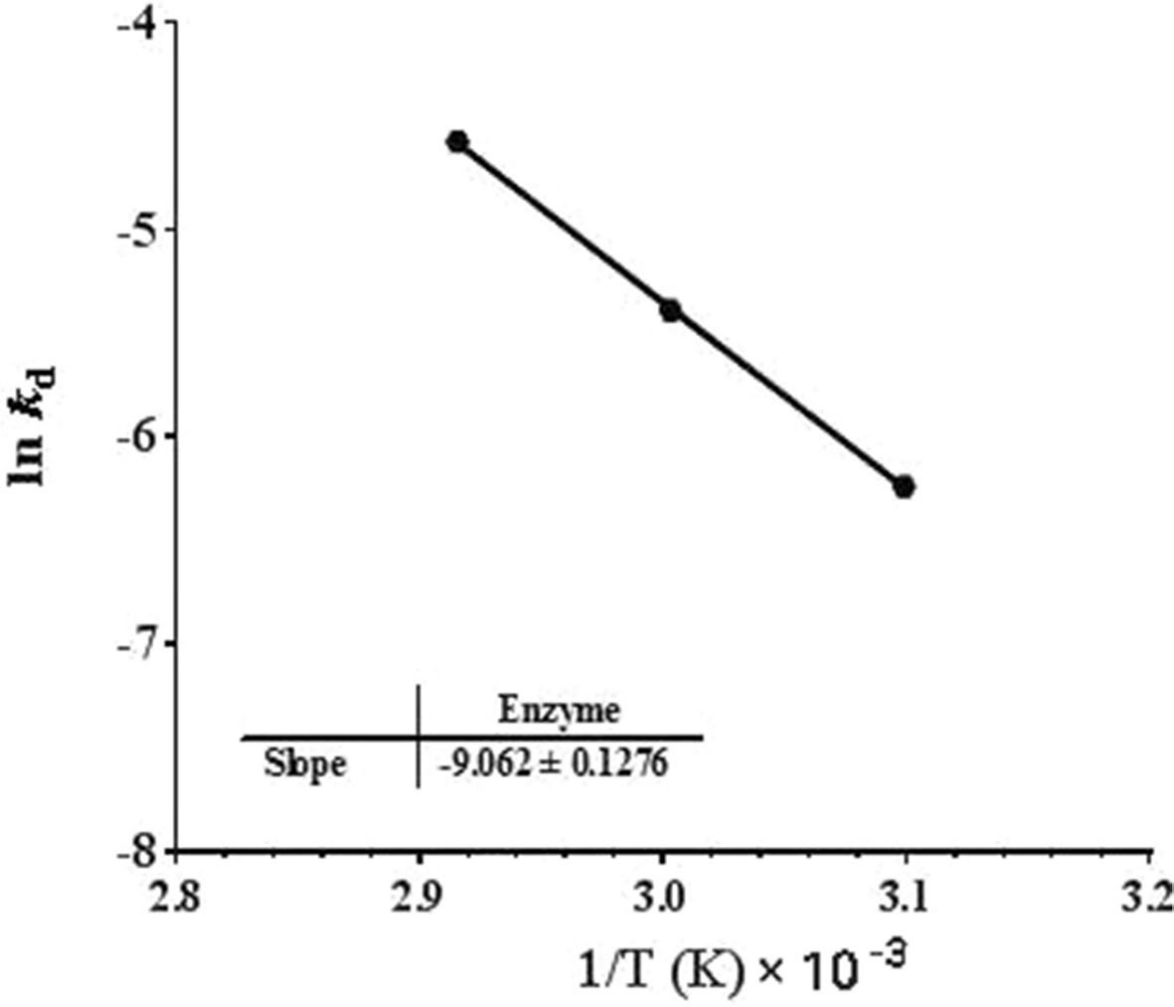
ln *K*_d_ against reciprocal of temperature

The results in Fig. 3a indicated that residual MCA decreased with increasing temperature and incubation time. The enzyme retained 81% activity after heating at 50 °C for 60 min. However, at the same incubation time (60 min), the enzyme retained 48% and 33% of its activity at 60 °C and 70 °C, respectively. The values of (*k*_d_), (*t*_1/2_), and (*D*) were calculated from Fig. 3b. The kinetic parameters for the purified *B. subtilis* MK775302 were recorded in Table 2. The deactivation energy for irreversible thermal inactivation (*E*_d_) was calculated from Arrhenius plot of Ln *K*_d_ against 1/*T* in Kelvin (Fig. 4).

**Table 2:** The kinetic parameters for the purified *B. subtilis* MK775302

Kinetic parameter	50 °C	60 °C	70 °C
*t*_1/2_ (min)	385	135.9	86.5
Thermal deactivation constant (*k*_d_) (min)	1.8 × 10^−3^	5.1 × 10^−3^	8.01 × 10^−3^
*(D)* value min	1279.4	451.6	287.8
Activation energy *E*_a_ (kJ/mole)	47.77		
Deactivation energy for irreversible thermal inactivation *E*_d_ (kJ/mole)	173.5

#### Effect of substrate concentration

**Fig. 5:**
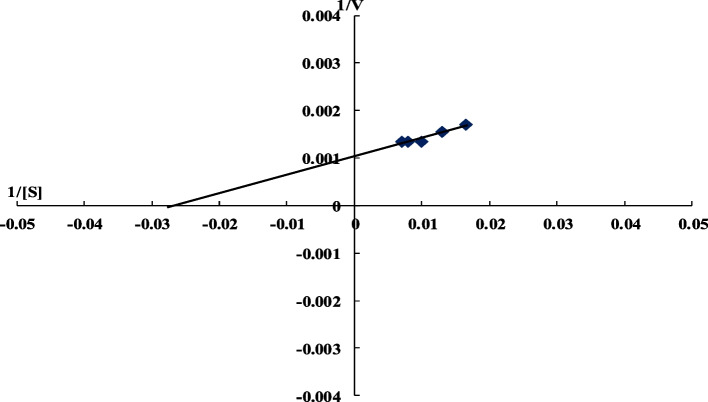
Lineweaver–Burk plot

MCE activity was assayed at different milk concentrations (60–140 mg/ml) at the optimum assay conditions. The gradual increase in substrate concentration caused an increase in enzyme activity. Maximal enzyme activity was obtained at a milk concentration of 120 mg/ml; above this concentration, the enzyme activity remained constant. *Km* and *Vmax* values of *B. subtilis* MK775302 MCE were calculated from the Lineweaver–Burk plot (Fig. 5).

#### Effect of NaCl on MCA of the partially purified enzyme

**Fig. 6:**
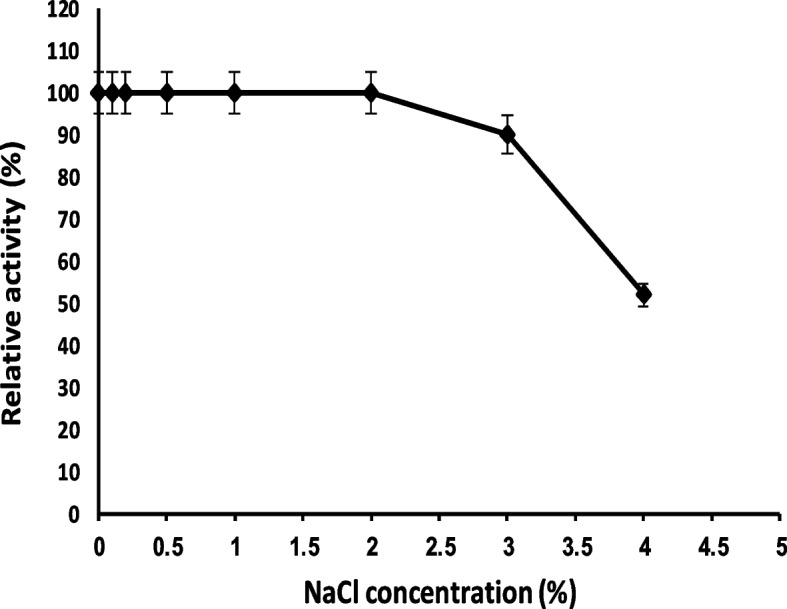
Effect of NaCl on *B. subtilis* MK775302-purified MCE

As illustrated in Fig. 6, the enzyme retained 100% activity at concentrations from 0.1 to 2% NaCl. At higher concentration of NaCl, MCA was decreased.

### Comparing the properties of the produced cheese samples

#### The moisture content

**Fig. 7:**
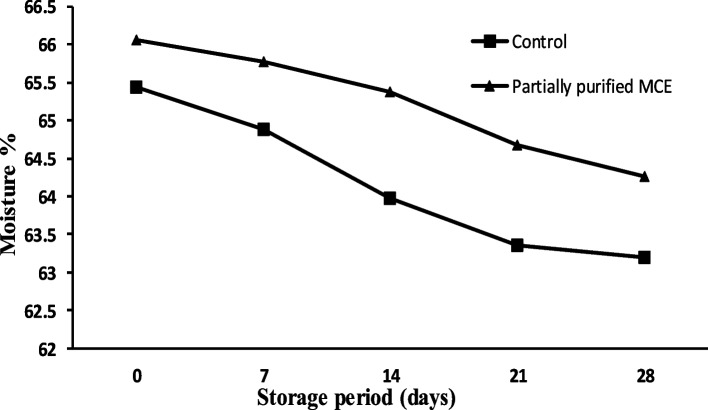
Moisture content of cheese samples during storage

The moisture contents of white soft cheese made from commercial calf rennet (control) and the partially purified MCE are shown in Fig. 7. The moisture contents of fresh cheese samples were 65.44 and 66.06% for the control calf rennet and partially purified enzyme cheese, respectively. The partially purified MCE has slightly higher moisture contents; however; they both lay in the same range.

#### Total acidity (TA %)

**Fig. 8:**
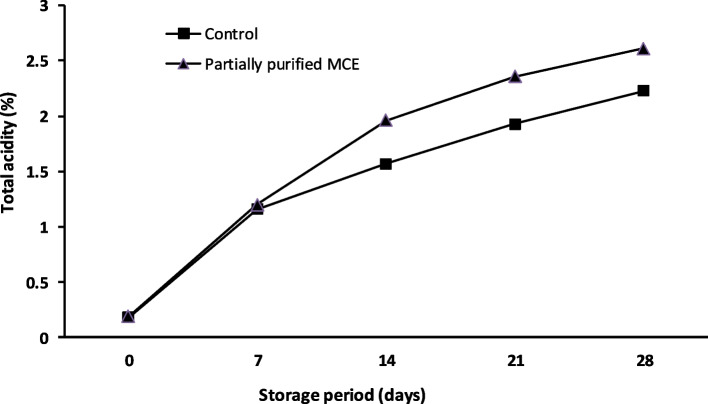
Total acidity of cheese samples during storage

The total acidity (TA) of white soft cheese made from commercial calf rennet (control) and purified *B. subtilis* MK775302 MCE are presented in Fig. 8. It was obvious that TA of fresh UF-white soft cheese samples were 0.18 and 0.19% for control and purified enzyme cheeses, respectively. During the storage, the TA was increased from 0.18 to 2.23% and from 0.19 to 2.61% for the control and the partially purified enzyme cheese, respectively.

#### Total nitrogen (TN %)

The total nitrogen (TN) contents in both cheese samples (Supplementary Fig. 1) were significantly increased (*P* ≤ 0.05) during the storage period**.** The partially purified enzyme cheese had higher TN content than the control treatment.

#### Soluble nitrogen (SN or WSN %)

The SN was determined in both experimental UF white soft cheese treatments during a storage period of 28 days at 5 °C (Supplementary Fig. 2). In fresh cheeses samples, SN contents were 0.15 and 0.21% for the control and the partially purified enzyme cheese samples, respectively. After 28 days, SN content was increased in both treatments; however, the partially purified enzyme cheese had higher SN content than the control treatment. It was observed that in both cheese treatments, the SN content was significantly increased (*P* ≤ 0.05) during the storage period.

#### Total volatile fatty acids content (TVFA)

The TVFA was determined in UF-white soft cheese with control calf rennet and the partially purified enzyme during a storage period of 28 days at 5 °C (Supplementary Fig. 3). The obtained results indicated that the rate of accumulation of TVFA significantly increased (*P* ≤ 0.05) with increasing the time of storage in both treatments. The values of TVFA in fresh samples were 18.34 and 19.64 ml of 0.1 N NaOH/ 100 g cheese for the control and purified enzyme cheeses, respectively. The values of TVFA after 4 weeks were 91.98 and 100.45 ml of 0.1 N NaOH/ 100 g cheese for the same treatments.

#### Textural profile analysis (TPA)

This includes hardness, gumminess, cohesiveness, and chewiness. The results of these different parameters are presented in Table 3. The hardness of both soft cheese samples was affected by the coagulants and the storage period. As indicated in Table 3, the purified enzyme treatment exhibited slightly lower hardness than the control treatment (calf rennet). So, the cheese made from the purified MCE has a softer (creamy) texture than that manufactured by using commercial calf rennet. During the storage period (from 7 to 28 days), the cheese hardness was gradually and significantly (*P* ≤ 0.05) increased in both treatments (control and purified enzyme coagulants).

**Table 3:** Textural profile analysis of soft cheese treated with different coagulants during ripening

**Textural properties**	**Ripening period (day)**	**Treatments**	
		**Calf rennet (control)**	**Partially purified MCE**
**Hardness** **N**	**0**	2.10	1.60
	**7**	3.90	2.20
	**28**	4.00	3.80
**Cohesiveness** **(B/A area)**	**0**	0.768	0.736
	**7**	0.674	0.737
	**28**	0.652	0.491
**Springiness** **Mm**	**0**	0.809	0.695
	**7**	0.752	0.797
	**28**	0.694	0.659
**Gumminess** **N**	**0**	1.613	1.178
	**7**	2.610	1.621
	**28**	2.630	2.357
**Chewiness** **N/mm**	**0**	1.305	0.818
	**7**	1.879	1.292
	**28**	1.912	1.553

*N* Newton, *mm* Millimeters, *N/mm* Newton/ml

The purified enzyme cheese treatment exhibited lower gumminess than the control cheese (Table 3). The gumminess was affected by the coagulant and the storage period. In general, during the storage period (28 days), gumminess had a tendency to increase in both treatments. The chewiness of soft cheese samples from both treatments was increased during the storage. The purified enzyme cheese exhibited lower chewiness than the control treatment.

### Sensory evaluation

**Fig. 9:**
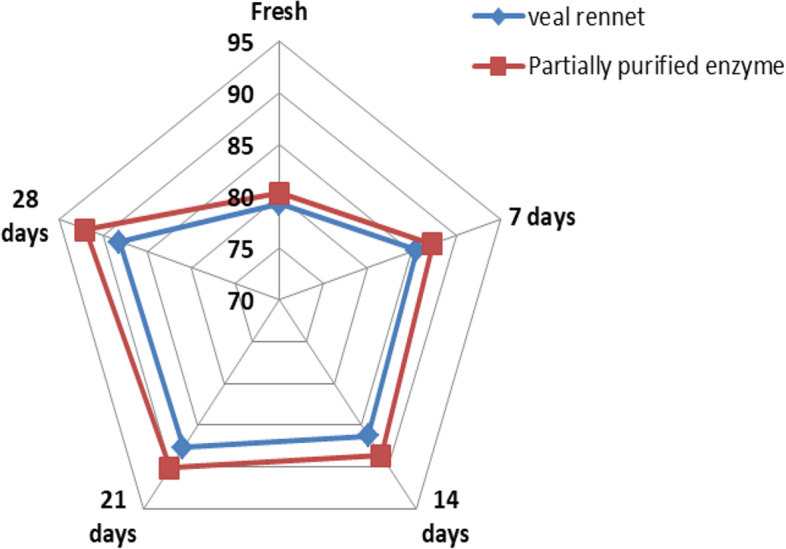
Total sensory evaluation of cheese samples of veal rennet (calf rennet) and partially purified enzyme during storage

As indicated in Table 4, the cheese produced from the partially purified *B. subtilis* MK775302 MCE possessed higher appearance, texture, and flavor scores than the commercial calf rennet cheese. The results in Fig. 9 showed that the total acceptability score of both cheese treatments was gradually increased by increasing the storage period. After 28 days, the total acceptability scores of cheese samples were 88.11 and 92 points for the control and purified enzyme cheese, respectively.

**Table 4:** Sensory evaluation of white soft cheese samples during ripening

Organoleptic properties	Storage period (days)	Calf rennet cheese	Partially purified enzyme cheese
Appearance (10)	0	6.80	6.33
	7	7.92	8.61
	14	8.35	8.71
	21	8.26	8.79
	28	8.23	9.05
Body and texture (40)	0	33.19	33.88
	7	34.49	35.22
	14	34.78	36.00
	21	35.70	36.52
	28	36.03	37.95
Flavor (50)	0	39.26	40.11
	7	43.13	43.51
	14	43.17	43.90
	21	43.70	44.83
	28	43.85	45.00
Total score (100)	0	79.25	80.32
	7	85.54	87.34
	14	86.30	88.61
	21	87.66	90.14
	28	88.11	92.00

## Discussion

The most active enzyme fraction with 5.6-fold purification was obtained at 50% acetone concentration. El-Bendary et al. [24] also obtained the most active fraction of MCE from *Bacillus sphaericus* NRC 24 with 50% acetone concentration but with only twofold purification. Many authors had reported that microbial MCE was better precipitated using acetone in comparison with ammonium sulfate [25, 26].

The optimum temperature (70 °C) is similar to the optimal temperature of MCE produced from *Bacillus thuringiensis kurstaki* [27] and higher than those reported for MCE from other microorganisms (60 °C for MCE from *Penicillium oxalicum*; 55 °C for MCA from *Bacillus sphaericus* and *Bacillus licheniformis*) [24, 28, 29]. It was reported that the optimal temperature of MCEs should be in the range of 35–75 °C [29–31]. In our study, the optimal temperature of *B. subtilis* MK775302 MCE was recorded in the range which makes it convenient for commercial utility. The decrease of activity at higher temperatures might be due to denaturation of MCE by heat. The activation energy (*Ea*) was calculated as 47.77 kJ/mol from the slope of Arrhenius plot (Fig. 1b). This *Ea* value is much lower than that calculated for protease from *Aspergillus fumigatus* (62 kJ/mol) [32]. However, lower values of *Ea* were calculated by other authors (22.99 kJ/mol for *Bacillus subtilis* MTCC 10,422 MCE; 29.27 kJ/mol for *Bacillus subtilis* KU710517 MCE) [33, 34].

The maximum MCA was observed at pH 5.0. The activity decreased at higher pH which might be due to the stability of casein micelles at high pH which led to a decrease in MCA [35]. Many authors have also observed a drop in MCA at high pH values [11, 36]. This high activity at acidic pH is extremely preferable for industrial applications. A similar optimum pH value was reported by other authors [37–39].

The results of thermal stability at 50, 60, and 70 °C are higher than those reported by other authors. For example, the MCE produced from *Bacillus licheniformis* BL312 completely lost its activity at 60 °C after 30 min. [29]. The MCE from *Bacillus subtilis* MTCC 10,422 retained 46% activity after 30-min incubation at 60 °C [33]; MCE produced from *Bacillus subtilis* KU710517 retained about 73 and 47% of enzyme original activity after heating for 15 min at 55 and 60 °C, respectively [40], and MCE from *Bacillus amyloliquefaciens* was completely deactivated when incubated at 55 °C for 20 min [29]. The stability of milk clotting enzymes at high temperatures for long time is of great advantage for their application in the cheese industry [33]. The calculated *t*_1/2_ values at 50, 60, and 70 °C were 385, 135.9, and, 86.5 min, respectively. These *t*_1/2_ values are much higher than those calculated for other MCE (*Aspergillus fumigatus* protease had *t*_1/2_ values of 65, 34, and 14 min at 50, 60, and 70 °C, respectively, and MCE from *Bacillus subtilis* MTCC 10,422 had *t*_1/2_ values of 43.9, 21, and 5.2 min at 50, 60, and 70 °C, respectively) [32, 33]. It was obvious that when the temperature was increased, the *t*_1/2_ and *D*-value were decreased; however, the first-order thermal deactivation rate constants (*K*_d_) were increased [33, 41, 42].

Maximal enzyme activity was obtained at a milk concentration of 120 mg/ml; above this concentration, the enzyme activity remained constant. This could be because of the substrate inhibition phenomenon, in which the enzyme molecules become fully saturated with substrate and all active sites are occupied, resulting in no further increase in activity. These observations were previously reported for other enzymes [43]. The *Km* is the substrate concentration required to produce half maximum velocity. The *Km* value affords information about the affinity between the enzyme and its substrate as the greater enzyme affinity has the lower *Km* value. From the Lineweaver–Burk plot (Fig. 5) the *Km* and *Vmax* values of *B. subtilis* MK775302 MCE were calculated as 36 mg/ml and 833 U/ml, respectively. This* Km* value is much lower than that reported by Abdel-Rahman et al. (2018) for milk clotting enzyme from *Pleurotus albidus* (*Km* was 0.2 g/ml, and Vmax value was 5000 U/ml) [44]. So, the enzyme in the present study had a higher affinity to the substrate than the enzyme from *Pleurotus albidus*. On the other hand, a lower *Km* value was reported for milk clotting enzyme produced by *Bacillus sphaericus* (5 mg/ml) [24]. For milk clotting enzyme produced by *Aspergillus niger*, *Km* value was 1.02 mM, and *Vmax* was 2.2 µmol/min [45]. *Aspergillus candidus* produced a milk clotting enzyme with *Km* and *Vmax* values of 0.059 mg/ml and 10.3 mmol/ml/s, respectively [46].

Sodium chloride plays a significant role in cheese ripening, controlling microbial growth, and texture of cheese [8, 47]. The MCA was decreased at NaCl concentration above 2%. Many authors also had observed a drop in MCA at high concentrations of NaCl [11, 16, 29]. High concentrations of NaCl could cause enzyme denaturation by salting-out process. Sodium chloride also could cause a decrease in MCA through some conformational changes in casein [8, 29]. So, the purified MCE from *B. subtilis* MK775302 is suitable for the production of specific cheese types such as low-salted white soft cheese and cheddar cheese.

The moisture contents of fresh cheese samples were 65.44 and 66.06% for the control calf rennet and the partially purified *B. subtilis* MK775302 MCE, respectively. Our observations are in harmony with those observed by Abou Ayana et al. [48] for calf rennet and *Mucor mucedo* MCE cheese. The moisture contents in both samples were decreased during the storage period (28 days) which might be due to increasing the acidity during storage. It is well known that the higher acidity in the milk decreases the moisture content [49, 50]. Other authors also found that the moisture content was decreased in ripened Danbo cheese [51].

The white soft cheese made from the partially purified MCE had higher TA than control cheese, and this might be due to the accumulation of lactose degradation products such as lactic acid and volatile fatty acids [7, 48, 51]. During the storage period, TA increased significantly (*P* ≤ 0.05), and this might be due to lactose fermentation in the presence of starter cultures which results in the production of some acidic compounds and might be also due to the degradation of protein and fat [11, 48, 50–52].

The total nitrogen (TN) contents in both cheese samples were significantly increased (*P* ≤ 0.05) during the storage period which might be owing to the decrease in the moisture contents. Similar observations were reported by other authors [53, 54].

The soluble nitrogen (SN) is considered an important index for cheese ripening as it reveals the rate of protein degradation [55]. The breakdown of protein is directly related to the texture and flavor during the ripening of cheese [56]. The partially purified enzyme cheese had higher SN content than the control treatment in both fresh cheese and after 28-day storage samples. This might be due to the high rate of proteolysis caused by proteases released from the partially purified MCE. It was observed that in both cheese treatments, the SN content was significantly increased (*P* ≤ 0.05) during the storage period. This might be due to increasing the proteolysis rate throughout the storage period. Similar observations were reported by other authors [39, 52].

The rate of TVFA accumulation increased with increasing the storage time in both control and partially purified enzyme treatments. Other authors also noticed a gradual increase in TVFA contents during the storage period which might be due to increasing the lipolysis rate [57]. It was clear that cheese made by the purified *B. subtilis* MCE had more volatile flavor compounds compared to the control, and this could be due to the high lipolytic activity of the coagulant. This was consistent with the results of other authors [39, 58].

The partially purified enzyme treatment exhibited slightly lower hardness than the control treatment due to variation in SN contents according to the coagulant. So, the cheese made from the purified MCE has a softer (creamy) texture than that manufactured by calf rennet. During the storage period, the cheese hardness was gradually increased in both treatments, and this might be due to the negative correlation between the moisture contents and hardness. Abd El-Salam et al. also observed an increase in the hardness in all cheese treatments with progressing the storage period [50]. Changes in hardness might be also due to the changes in the total solids content of cheese samples as a close relation was obvious between hardness and the total solids.

Gumminess was affected by the coagulant and the storage period. The chewiness of soft cheese samples from both treatments was increased during the storage. The purified enzyme cheese exhibited lower chewiness than the control treatment. This difference in textural properties between samples might depend on the type of rennet used and the difference in free amino acids and fatty acid contents. Generally, the changes in texture profile properties during storage might be caused by proteolysis, glycolysis, lipolysis, and changes in pH [7].

The total acceptability score of the partially purified enzyme cheese and calf rennet cheese was gradually increased by increasing the storage period. The cheese produced from the partially purified MCE possessed higher appearance, texture, and flavor scores than the calf rennet cheese. This may be due to variation in the free fatty acids and amino acid contents which have a significant effect on the flavor. Many previous studies reported that cheese made from MCE derived from *Bacillus* usually has unsuitable sensory due to the high bitter peptides contents [59]. However, in this study, cheese made by the partially purified *B. subtilis* MCE had no bitterness even after 28 days of storage. It also had higher volatile flavor compounds and higher total acceptability score than the commercial calf rennet cheese. For these reasons, the partially purified *B. subtilis* MK775302 MCE produced in our study is recommended as a promising rennet substitute.

## Conclusions

The thermal stability of the partially purified *B. subtilis* MK775302 MCE at high temperatures for prolonged time gave it a great advantage for suitability in cheese industry. Compared with the commercial calf rennet, the partially purified *B. subtilis* MK775302 MCE produced UF white soft cheese with improved texture and flavor and a higher acceptability score. Therefore, it is recommended as an excellent coagulant that can replace calf rennet in the production of white soft cheese with improved properties. It also can solve the problem of low availability of calf rennet in the local markets.

## Supplementary Information

**Additional file 1: Supplementary Fig. 1.** Total nitrogen (TN) content of cheese samples during storage. **Supplementary Fig. 2.** Soluble nitrogen (SN) content of cheese samples during storage. **Supplementary Fig. 3.** Total volatile fatty acids (TVFA) of cheese samples during storage.

## Data Availability

Not applicable.

## Abbreviations

MCE: Milk clotting enzyme
MCA: Milk clotting activity
*E*a: Activation energy
*t*_1/2_: Half-life time
*V*_ma__x_: Maximum enzyme velocity
*K*_m_: Michaelis constant
TN: Total nitrogen
SN: Soluble nitrogen
WSN: Water-soluble nitrogen
TA: Titratable acidity
TVFA: Total volatile fatty acids
