# Supplementary material for: The catalytic and kinetic characterization of Bacillus subtilis MK775302 milk clotting enzyme: comparison with calf rennet as a coagulant in white soft cheese manufacture
Source: J Genet Eng Biotechnol. 2023 May 17;21:61. doi: 10.1186/s43141-023-00513-w (PMC10192502; doi:10.1186/s43141-023-00513-w)
Supplement: Supplementary file 1 — Additional file 1: Supplementary Fig. 1. Total nitrogen (TN) content of cheese samples during storage. Supplementary Fig. 2. Soluble nitrogen (SN) content of cheese samples during storage. Supplementary Fig. 3. Total volatile fatty acids (TVFA) of cheese samples during storage. [file 43141_2023_513_MOESM1_ESM.docx]

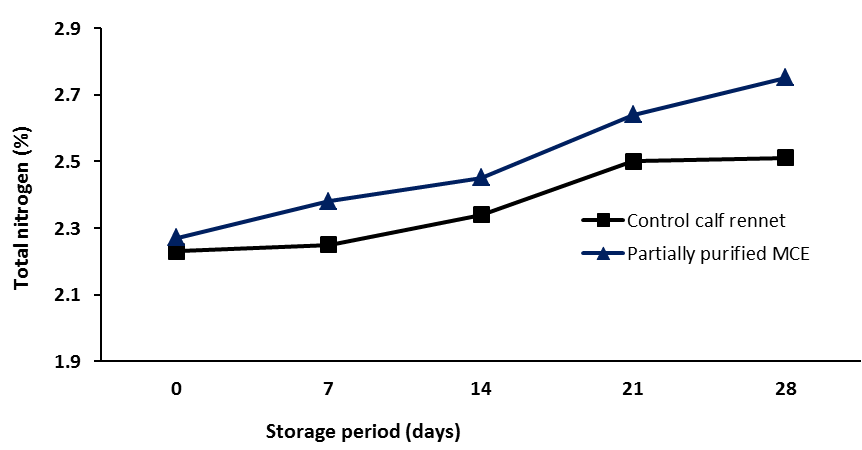


**Supplementary Fig. 1** Total nitrogen (TN) content of cheese samples during storage


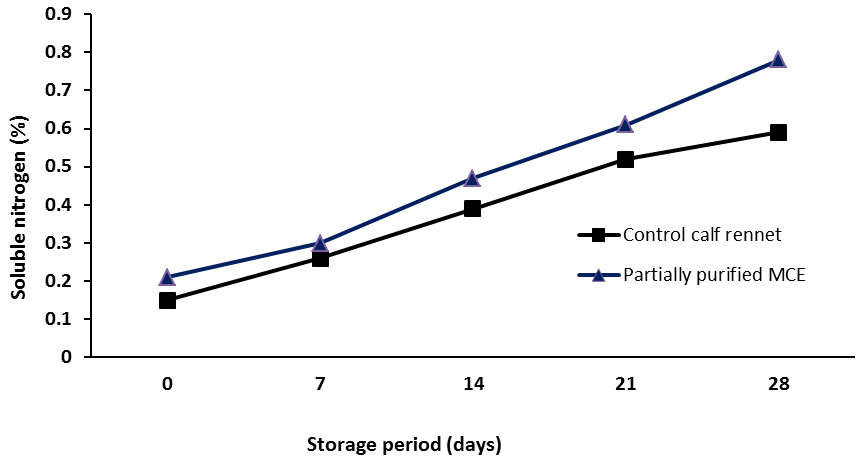


**Supplementary Fig. 2** Soluble nitrogen (SN) content of cheese samples during storage


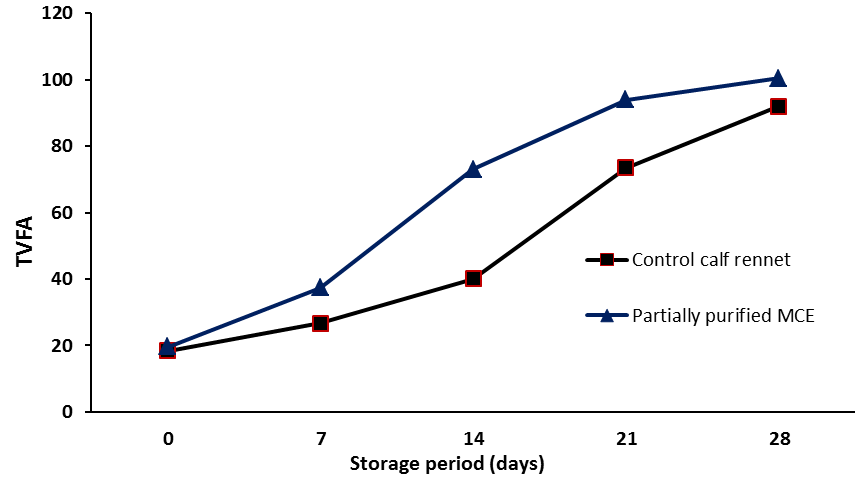


**Supplementary Fig. 3** Total volatile fatty acids (TVFA) of cheese samples during storage
